# A Wearable Electrochemical Gas Sensor for Ammonia Detection

**DOI:** 10.3390/s21237905

**Published:** 2021-11-27

**Authors:** Martina Serafini, Federica Mariani, Isacco Gualandi, Francesco Decataldo, Luca Possanzini, Marta Tessarolo, Beatrice Fraboni, Domenica Tonelli, Erika Scavetta

**Affiliations:** 1 Dipartimento di Chimica Industriale “Toso Montanari”, Università di Bologna, Viale del Risorgimento 4, 40136 Bologna, Italy; martina.serafini6@unibo.it (M.S.); domenica.tonelli@unibo.it (D.T.); erika.scavetta2@unibo.it (E.S.); 2 Dipartimento di Fisica e Astronomia, Università di Bologna, Viale Berti Pichat 6/2, 40127 Bologna, Italy; francesco.decataldo2@unibo.it (F.D.); luca.possanzini2@unibo.it (L.P.); marta.tessarolo3@unibo.it (M.T.); beatrice.fraboni@unibo.it (B.F.)

**Keywords:** wearable sensor, electrochemical gating, gas sensor, ammonia detection, PEDOT:PSS

## Abstract

The next future strategies for improved occupational safety and health management could largely benefit from wearable and Internet of Things technologies, enabling the real-time monitoring of health-related and environmental information to the wearer, to emergency responders, and to inspectors. The aim of this study is the development of a wearable gas sensor for the detection of NH_3_ at room temperature based on the organic semiconductor poly(3,4-ethylenedioxythiophene) (PEDOT), electrochemically deposited iridium oxide particles, and a hydrogel film. The hydrogel composition was finely optimised to obtain self-healing properties, as well as the desired porosity, adhesion to the substrate, and stability in humidity variations. Its chemical structure and morphology were characterised by infrared spectroscopy and scanning electron microscopy, respectively, and were found to play a key role in the transduction process and in the achievement of a reversible and selective response. The sensing properties rely on a potentiometric-like mechanism that significantly differs from most of the state-of-the-art NH_3_ gas sensors and provides superior robustness to the final device. Thanks to the reliability of the analytical response, the simple two-terminal configuration and the low power consumption, the PEDOT:PSS/IrOx Ps/hydrogel sensor was realised on a flexible plastic foil and successfully tested in a wearable configuration with wireless connectivity to a smartphone. The wearable sensor showed stability to mechanical deformations and good analytical performances, with a sensitivity of 60 ± 8 μA decade^−1^ in a wide concentration range (17–7899 ppm), which includes the safety limits set by law for NH_3_ exposure.

## 1. Introduction

Among hazardous gaseous compounds causing severe health issues, ammonia has good warning properties thanks to its pungent smell and to the low odour threshold of our olfactory system [1]. However, adaptation or olfactory fatigue upon routine exposure makes NH_3_ inhalation a bio-threat for operators working in many industrial contexts, including agriculture, the fertilizer and automotive industries, animal breeding, and industrial refrigeration [2]. In this regard, the fast-evolving field of wearable electronics may be a great resource for future occupational safety and health management strategies, wirelessly providing health-related and environmental information in real time to the wearer, emergency responders, and inspectors.

Due to the high solubility of NH_3_, contact with its vapours immediately causes irritation to eyes, mucous membranes, and the whole respiratory system, while exposure to high concentrations can lead to life-threatening conditions, such as pulmonary edema, airway destruction, and respiratory failure. The exposure limit is set to 25 ppm averaged over an eight-hour workday and 35 ppm for short-term exposure [3]. In order to perform routine sampling, in situ continuous monitoring, real-time detection of eventual gaseous hazard, and the prompt following of safety precautions, the consolidated analytical techniques, such as gas chromatography, are impractical due to the large size of the equipment and slow response time [4]. Conversely, miniaturized dosimeters and belt-worn analytical tools are available to the market and mainly target the real-time monitoring of air pollution, indoor air quality, aerosol exposure, and detection of biothreat agents. Despite portability and robustness, such devices rely on sophisticated optical detectors that profoundly impact on the final cost of the product [5]. Electronic noses offer a more innovative and biomimetic approach, where arrays of solid-state receptors are associated with pattern recognition algorithms, leading to the elaboration of complex olfactory maps (i.e., odour fingerprints) after a training period. Recently, proof-of-concept studies on wearable electronic noses realised on plastic foils or textiles have been reported for human skin odour identification for biometric purposes [6,7], healthcare [8], analysis of volatile compounds in exhaled breath [6], and hazardous gas leakage detection in the surrounding environment [9,10]. The most widely investigated sensing configuration in this kind of technology is the chemiresistor, typically including metal oxide semiconductors (MOs) as sensing materials, which exhibit a change in conductivity in response to chemical stimuli originating from the sorption of gas molecules at the solid/gas interface [4]. MO-based gas sensors usually rely on either surface or bulk conductance effects, require high-temperature activation steps, and suffer from cross-sensitivity and limited environmental stability due to humidity variations and changes in chemisorbed O_2_ [2]. As an alternative to MOs, conducting polymers (CPs) and their composites have emerged among gas sensing materials for NH_3_ detection, mainly thanks to the unique ability of polyaniline (PANi) to interact with NH_3_ by acid-base doping mechanisms. In particular, the analyte de-dopes the protonated centres of the emeraldine salt by forming NH_4_^+^ ions, thus leading to an increase in the polymer film resistance. Starting from the first reports concerning PANi-based ammonia sensors operating at room temperature in the 1990s [11,12], significant research efforts have been conducted to improve the sensitivity, processability and environmental stability of PANi films, especially with the development of nanostructured, composite, and co-doped PANi-based transducers [4]. Moreover, thanks to inherent CP advantages, including lightweight, low cost, room temperature stability, flexibility, and easy processing from solutions and dispersions with respect to other bulk semiconductors, some examples of NH_3_ chemiresistive sensors have been designed in a wearable form-factor in recent years. Relevant examples include wearable sensors realised using PANi-coated ZnO nanosheets [13] and PANI-functionalized multiwalled carbon nanotubes (MWCNTs) [14,15] on fabric substrates and plastic fibers, graphene oxide/PANi nanospheres [16] and PANi-CeO_2_ nanocomposite [17] on plastic foils, and bacterial cellulose functionalised with co-doped PANi nanorods [18]. Flexible, CP-based NH_3_ gas sensors have also been obtained using nanostructures and composites of poly(3,4-ethylenedioxythiophene) (PEDOT) [19,20,21], as well as Polypyrrole (PPy) [22,23].

Despite chemiresistive sensors representing one of the most convenient transduction units in terms of simplicity, low cost, and compatibility with large-scale manufacturing, they only allow a single-type output and usually exhibit drift to external perturbations and limited selectivity. For these reasons, wearable gas sensors are facing significant technological challenges that limit their applicability to an initial stage of development. Conversely, the design of novel transducers with alternative sensing mechanisms could be an appealing strategy to overcome these limitations. In fact, the development of multivariable sensing platforms with sensor arrays based on diversified transduction mechanisms [24], as well as the introduction of innovative approaches inspired by the working principles of well-assessed analytical techniques, could largely improve the stability and reliability of emerging wearable gas sensors. 

Our group has recently developed a new approach inspired by the organic electrochemical transistor for the fabrication of sensors operating in an aqueous environment with a two-terminal structure typical of a chemiresistor [25,26,27,28]. The sensing strategy is based on the combination of a conductive polymer and a potentiometric responsive material, acting as the charge transport layer and the transducing element, respectively. Thanks to the intimate contact between these two components, the potentiometric transducer, either in the form of a thin film or discrete particles embedded in the polymer, generates an electromotive force that exerts a spontaneous gate action on the organic semiconductor in response to a variation of the analyte concentration. The result is the reversible modulation of the doping state of the conductive polymer, with the consequent variation of the current flowing across the film. This approach has recently been exploited for the design of a two-point probe, electrochemically gated sensors for Cl^−^ and pH detection, operating in wound exudate [28] and sweat [25,27], focusing on healthcare applications. 

The simple architecture, the low applied voltage, and the operation at room temperature suggest that this approach could be successfully applied to the design of wearable devices able to monitor hazardous gas concentration in the environment surrounding the wearer. In this work, we exploit the pH transducer based on PEDOT functionalised with iridium oxide particles (IrOx Ps), usually working in an aqueous environment, to detect gaseous NH_3_. Because the sensor must detect the analyte in a gas phase, the active material was coated with an agarose hydrogel film, which enables the transduction process to occur through an electrochemically gated mechanism triggered by pH variations. In particular, the diffusion of NH_3_ gas molecules causes pH changes within the hydrogel film, leading to a spontaneous gating of the PEDOT film conductivity mediated by the potentiometric pH transducer IrOx. As the response of electrochemical gas sensors can be affected by interfering species [29], the selectivity was also evaluated. Thanks to its light weight, its robustness, and its low power consumption, the sensor realised on a plastic substrate was successfully validated in a portable configuration, including handheld readout electronics, which was wirelessly connected to a smartphone application via Bluetooth for the real-time monitoring of NH_3_. 

## 2. Materials and Methods

### 2.1. Chemicals and Buffers

CLEVIOS^TM^ PH 1000 suspension (PEDOT:PSS) was purchased from Heraeus (Hanau, Germany). (3-glycidyloxypropyl)trimethoxysilane (GOPS), sodium dodecylbenzenesulfonate, potassium nitrate, potassium hydroxide, sodium hydroxide, nitric acid, boric acid, hydrochloric acid, sodium dihydrogen phosphate, agarose, ammonia (28–30%), glycerol (≥99.5%), methyl red, phenolphthalein, thymol blue, bromothymol blue, and methylene blue were purchased from Sigma Aldrich (St. Louis, MO, USA). Ethylene glycol (EG) was obtained from Carlo Erba (Milano, Italy). Iridium tetrachloride (IrCl_4_, powder) was purchased from Alfa Aesar (Ward Hill, MA, USA). Silicone elastomer and curing agent for preparation of PDMS were obtained from Sylgard (Dow Europe, Weisbaden, Germany). All chemicals were of reagent grade or higher.

### 2.2. Apparatus

The electrochemical deposition of the IrOx Ps was carried out in a single compartment, three-electrode cell using a potentiostat (CH Instrument 660 C). An aqueous saturated calomel electrode (SCE) and a Pt gauze were reference (RE) and counter electrode (CE), respectively. A combined glass electrode (Amel 411/CGG/12) connected to a pH meter (Amel instruments 338) was employed for pH measurements and potentiometric titrations. Gas sensing measurements were carried out with a Source-measure Unit (Keysight B2902A) and a portable sampler AirCube^®^ COM2 (Analitica Strumenti, Pesaro, Italy). IR-ATR spectra were obtained with a PerkinElmer FT-IR (Waltham, MA, USA) spectrometer Spectrum Two. Scanning Electron Microscope (SEM) images were obtained on Cr/Au-coated, freeze-dried hydrogel samples using the Cambridge Stereoscan 360 (Oxford Instruments, Abingdon, UK) instrument. 

### 2.3. Gas Sensors Fabrication

The Cr/Au (10/40 nm) tracks were thermally evaporated, as described elsewhere [28,30]. Then, CLEVIOS^TM^ PH1000 suspension was mixed with EG, dodecylbenzene sulfonic acid and GOPS in the following volumetric ratio, 93.75:5:0.25:1, sonicated for 10 min and filtered using 1.2 μm cellulose acetate filters (Sartorius, Gottingen, Germany). After air plasma cleaning (4 min, 15 Watt) the substrates, the PEDOT:PSS solution was spun cast on the masked substrates, obtaining a film with a thickness of about 600 nm. Afterwards, the devices were thermally annealed for 1 h at 140 °C to improve the film stability [31], and a mixture of PDMS-curing agent (9:1 *w*/*w*) was applied over the Au contacts to ensure electrical insulation. The PEDOT:PSS film was electrochemically functionalised with iridium oxide particles (IrOx Ps), following a procedure described elsewhere [28]. Briefly, 10% wt. NaOH solution was added dropwise to a 2 mM IrCl_4_ aqueous solution until the pH turned basic, giving a light-yellow colour. The solution was heated at 90 °C for 1 h, under stirring, and then fast cooled down in an ice bath. 3 M HNO_3_ was added dropwise until the solution pH turned acid. After 80 min stirring at room temperature, the dark-blue suspension containing IrOx Ps was aged at 4 °C and protected from light sources for 24 h. The suspension was employed as the electrolyte solution to carry out the electrodeposition of IrOx Ps. For this, a standard electrochemical cell was used where the polymer film of a two-terminal device was the WE, while a Pt gauze and a SCE were the CE and RE, respectively. The potential of the WE was scanned between 0 < E < 1 V vs. SCE for 100 cycles at 100 mV s^−1^. Finally, the resulting PEDOT:PSS/IrOx Ps film was coated with the agarose hydrogel, and the optimized procedure is described here. Agarose was dissolved in a 0.1 M KNO_3_ aqueous solution at 90 °C until a viscous and clear mixture was obtained with a final concentration of 0.7% wt. It was then removed from the hotplate and the NH_3_ aqueous solution was added to neutralise acid residues within the gel structure, reaching a final concentration of 90 mM. The PEDOT:PSS/IrOx Ps film was immersed in the still-warm mixture and left at room temperature until the hydrogel film formed spontaneously on top of the functionalised film. After that, the device was immersed in glycerol for 10 s and left at room temperature for 1 h. The same fabrication procedure was carried out on transparent, 125 µm thick Polyethylene napthalate (PEN) films to realise the flexible sensors.

### 2.4. Gas Sensing Setup and Measurements

A bench-size stripping system was built inside a lab fume hood, in order to obtain ammonia rich vapours of a controlled concentration from standardized aqueous solutions (Figure S1). The stripping system assembly and calibration are thoroughly described in the Supporting Information. Briefly, a pump for air sampling was used to apply a vacuum to the pipes connecting Drechsel bottles with D.I. water and NH_3_ aqueous solutions and drive the gas stream throughout a sensing chamber (flow rate of 2 L min^−^^1^). Humid air and gaseous NH_3_ streams were alternated using a three-way valve connected to the Drechsel bottles. The relative humidity of all humid air and NH_3_-containing streams was 57 ± 3%, and the sensor was always tested at room temperature (293 K). The stripping system was calibrated by means of a validated analytical method (see Figures S2–S4 and Table S1) [32]. Ammonia detection with the hydrogel-coated gas sensor based on PEDOT:PSS/IrOx Ps was carried out by placing the two-terminal device inside the detection chamber upon exposure to the gas stream. NH_3_-rich laboratory air streams (from 40 to 7899 ppm NH_3_) were delivered for 100 s and alternated to air flow until full baseline recovery, using a flow rate of 2 L min^−1^. For the measurements carried out without waiting for baseline recovery, the NH_3_-free air flow was delivered for a fixed time of 600 s. In all the experiments, a voltage difference of −200 mV was applied to the sensor and the generated current was measured vs. time. 

### 2.5. Statistical Analysis

Data are presented as mean ± standard deviation obtained from 3 independent calibrations (N = 3). The detection limit (LoD) was calculated as the x-axis intercept from the calibration plot. The quantification limit (LoQ) was instead calculated using the formula $\log\mathrm{LoQ}=K_{Q}*s_{b}/m$, with K_Q_, s_b_ and m being 10, the standard deviation of the blank signal, and the slope of the calibration curve. The steady state current recorded at the beginning of each experiment was taken as the baseline (blank signal). In contrast, the current recorded immediately before each exposition to NH_3_ was taken as the blank signal in the experiments carried out without waiting for the baseline recovery.

## 3. Results

### 3.1. Gas Sensor Design

The acid/base properties of NH_3_ were exploited to perform its detection in air streams, using a simple two-terminal pH sensor based on PEDOT:PSS and IrOx Ps. 

**Figure 1:**
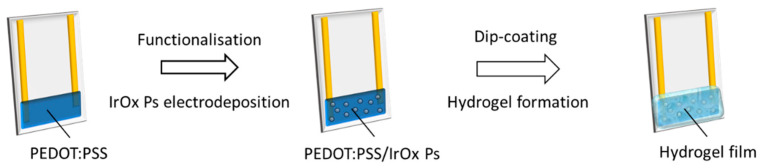
Schematic representation of the procedure employed for gas sensor fabrication.

A schematic representation of the procedure employed for sensor fabrication is reported in Figure 1 and can be divided into two steps: (i) functionalisation of the polymer film with IrOx Ps and (ii) hydrogel formation. The electrochemical deposition of IrOx Ps was carried out by scanning the potential applied to the PEDOT:PSS film deposited between the two metal contacts (0 < E < 1 V vs. SCE, at 100 mV s^−1^ for 100 cycles), using an aqueous suspension of IrOx Ps as the electrolytic solution in a standard three-electrode cell. This procedure, described and optimised elsewhere [28], leads to the embedding of around 500 nm diameter IrOx Ps in the PEDOT:PSS film and was shown to impart pH-sensing capabilities to the organic semiconductor. The as-obtained device is an electrochemically gated pH sensor, where the IrOx particles modulate reversible doping/de-doping processes in the polymer film following a potentiometric pH-mediated mechanism in aqueous solutions (Figure S5). It is noteworthy that the aqueous environment, in which the gas sensor operates, plays an active part in allowing the detection mechanism. First of all, it enables acids and bases dissociation to release H^+^/OH^−^ ions that change the solution pH, thus generating the chemical signal that is transduced by the sensor. Secondly, electroneutrality during the redox processes involving PEDOT^+^/PEDOT is maintained thanks to the cations exchanged between the electrolyte solution and the organic film, where the excess of negatively charged PSS^−^ must be compensated. Therefore, in order to make the PEDOT:PSS/IrOx Ps pH sensor usable for gas sensing, the electrochemical interface was further modified with an ionic hydrogel mimicking the aqueous environment and allowing the sensor to interact with the gaseous analyte. 

### 3.2. Optimization of the Hydrogel Composition

The formulation of the hydrogel was optimized to guarantee: (i) physico/chemical stability, (ii) reversible absorption of gaseous NH_3_ molecules, and (iii) the capability to promptly change its pH in response to the amount of absorbed NH_3_. Therefore, adhesion to the polymeric film and stability to humidity changes in air stream, concentration of the hydrogel precursor, and the pH of the hydrogel were systematically investigated. An agarose-based hydrogel was chosen for this study due to its biocompatibility and biodegradability [33], tuneable porosity, pore size distribution [34], facile preparation method, fast gelation time at room temperature, good mechanical match with the soft polymer film, and adherence to its surface. However, when placed inside the sensing chamber of the stripping system under real experimental conditions, the PEDOT:PSS/agarose interface rapidly dried during alternate exposure to laboratory air and saturated laboratory air. While stability to humidity variations is an essential requirement for a gas sensor operating in real life conditions, water-loss-induced instability is an intrinsic problem of hydrogels. To address this issue, water molecules in the hydrogel structure can be partially replaced by hygroscopic polyols, such as glycerol, via a simple solvent-exchange method. It has been shown that excellent moisture retention and cryoprotective properties are obtained thanks to the formation of hydrogen bonds between the water molecules and the hydroxyl groups of the polyol in the hydrogel [35,36,37]. Therefore, agarose hydrogel-coated PEDOT:PSS (PEDOT:PSS/agarose) and glycerol-treated (PEDOT:PSS/agarose/gly) films were tested upon the application of a potential difference between the two terminals while exposed to humidity changes in air streams. As evident from Figure S6, the glycerol treatment significantly improves the stability of the recorded current, keeping the hydrogel wet and adherent for up to 3 weeks. The fundamental role of glycerol was also confirmed by comparing the % weight loss ((w_0_ − w_i_)/w_0_), with w_0_ and w_i_ being the initial and i-th day weight, respectively) of the treated and untreated hydrogel films, which were stored in a Petri dish at ambient conditions. As a result, the glycerol-treated film still retained 88% of its initial weight after 6 days, while the glycerol-free hydrogel film showed a dramatic 98% weight loss in the two days following the synthesis.

**Figure 2:**
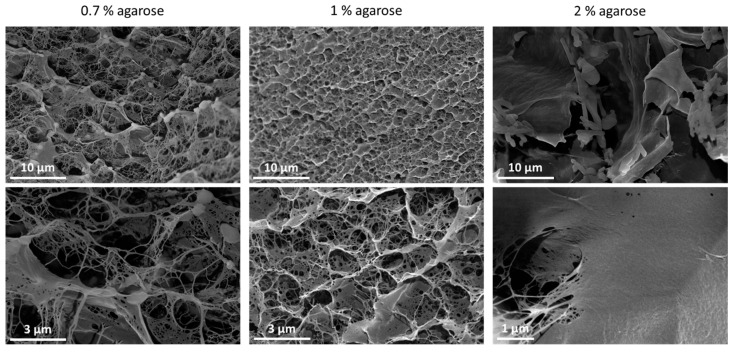
SEM images of 0.7%, 1%, and 2% agarose hydrogels.

Afterwards, the pH and the composition of the hydrogel were optimised, specifically targeting the gaseous NH_3_ detection with the two-terminal pH sensor placed in the detection chamber of the bench-size stripping system (Figure S1). For this, it is worth keeping in mind that NH_3_ gas molecules, once having reached the hydrogel/air interface, must first diffuse inside the hydrogel, where solvated NH_3_ produces OH^−^ according to its basic dissociation equilibrium:

(1) $$NH_{3}+H_{2}O⇄NH_{4}^{+}+OH^{-}$$

eventually changing the pH of the surrounding aqueous environment. Upon removal of the NH_3_-rich air stream by delivering humid air to the detection chamber, NH_3_ molecules should diffuse back from the bulk hydrogel film to the hydrogel/air interface until desorption, with the consequent restoration of the pristine pH value. In a preliminary experiment, two PEDOT:PSS/IrOx Ps films were modified with gly-treated hydrogels prepared with 2% agarose in 1 mM phosphate buffer (PBS, pH 7.00) and 0.1 M KNO_3_. When tested for NH_3_ detection, the presence of PBS caused prolonged current drift that we attribute to the buffered environment hindering the expected pH variations caused by reaction (1), thus impeding the transduction mechanism based on the pH sensing (Figure S7a). In contrast, the use of 0.1 M KNO_3_ improved the quality of the sensor response as the pH of the hydrogel could freely vary following the increase in [NH_3_]_g_ in the ammonia stream (Figure S7b). However, after removing NH_3_ from the gas stream, the current did not recover its starting value, suggesting a slow kinetics of NH_3_ molecule desorption at the hydrogel/air interface. With the aim of improving gas permeability across the gel, a screening study was carried out to lower the concentration of the hydrogel precursor and to obtain a structure with larger pores. In fact, pore size has been reported to decay with the concentration of agarose due to an increased rate of nucleation and a tighter packing of the chains [38,39]. A set of solutions was made up with an agarose concentration ranging from 0.2 to 2% in 0.1 M KNO_3_, and the minimum agarose concentration leading to successful gelation was 0.7% (Figure S8a). Figure 2 reports the morphology of 0.7%, 1%, and 2% agarose hydrogels analysed by SEM after freeze-drying. 

In accordance with other literature reports [34,40], the microstructure clearly reveals a mesh-like network of interconnected pores showing two main size distributions, whose diameter and wall thickness display up to a 20% decrease, passing from 0.7 to 1% agarose concentration. In contrast, the 2% agarose concentration leads to a massive tightening of the meshes, which seem to completely merge throughout extended regions of continuous hydrogel film. Here, the average diameter of the larger pores is five times smaller than those of the most diluted sample (0.7%). Due to its open structure with thicker walls, the 0.7% agarose hydrogel, showing pore diameters of (2.1 ± 0.9) × 10^2^ nm and (1.1 ± 0.2) × 10^3^ nm, respectively, with wall thicknesses of (1.0 ± 0.4) × 10^2^ nm, was chosen for all the further investigations. 

**Figure 3:**
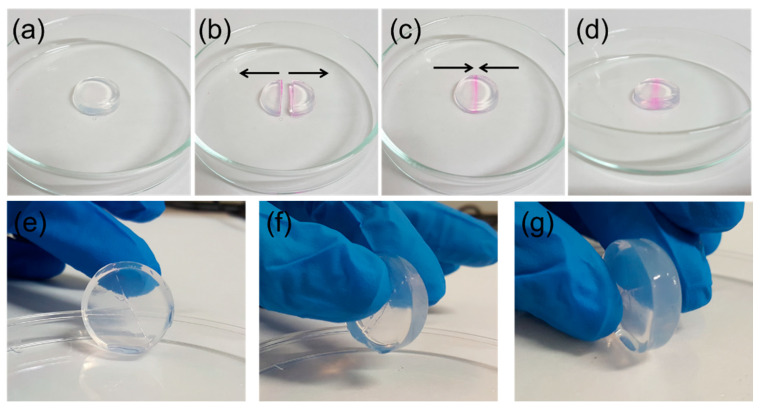
Self-healing properties in a free-standing, 0.7% agarose hydrogel cylinder. From (**a**–**c**): pristine hydrogel cylinder, cutting event with separation of the two halves, and reassembly of the two halves. (**d**) Self-healing waiting phase of the hydrogel. From (**e**–**g**): handling of the completely self-healed hydrogel cylinder.

The mechanical robustness of the 0.7% agarose hydrogel was therefore assessed. Remarkably, the as-synthesised hydrogel showed self-healing properties, which were demonstrated by cutting a free-standing cylinder with a scalpel and verifying its capability to autonomously recover the initial integrity (Figure 3). The edge of the blade was stained with a pink dye solution to highlight the cut location. As a major component of the gas sensor, the introduction of self-healing functions in the hydrogel may be an interesting strategy to improve the device’s durability against mechanical damage. Full healing was observed in about 10 min after the cutting event. 

The response of a sensor realised with the hydrogel containing 0.7% agarose in 0.1 M KNO_3_ after gly treatment is reported in Figure S8b. In this case, the recorded signal showed a tendency to recover the initial value upon ammonia stream removal, suggesting that the sensing process is partially reversible. The sharpness and intensity of the recorded signal were then evaluated by testing the same device during repeated exposure to high NH_3_ concentrations (from 395 to 7899 ppm), as shown in Figure S9. It was noticed that only after several exposition periods did the peak intensity in response to the equimolar NH_3_ concentrations become repeatable, and the recovery time depended on the analyte concentration, suggesting an initially hindered pH variation within the hydrogel. This hypothesis was qualitatively confirmed upon visual inspection of pH dyes containing agarose hydrogels spots deposited on a glass slide, showing that the pristine hydrogel had an acidic pH, with the consequent detrimental effect on NH_3_ detection (Figure S10). The reason could be ascribed to the presence of either agaropectin residues or sulfate groups linked to the agarose structure, depending on the separation method employed for the agarose production and the seaweed from which agar is extracted [41]. Therefore, increasing amounts of a NH_3_ solution were added to the agarose formulation (corresponding to 0.4, 1, 10, and 90 mM) to adjust the spontaneous pH of the hydrogel and avoid the neutralisation of the first NH_3_ gas molecules interacting with the acidic hydrogel environment. The real-time responses obtained using 0.4 and 100 mM NH_3_ are reported in Figure S11 and the performances of the four devices are compared in Figure S12. While the sensitivity is almost unaffected by the different NH_3_ loadings, the limit of detection (LoD) significantly decreases with the raise of [NH_3_] in the hydrogel. For these reasons, the highest NH_3_ loading was chosen. The IR spectra of the agarose hydrogels were recorded following each optimisation step and are discussed in SI (Figure S13). The spectroscopic characterisation demonstrates that the chemical structure of the agarose/gly hydrogel clearly results from the combination of its major components (water, agarose, and glycerol). Despite the final inclusion of NH_3_ within the hydrogel, the spectrum remains unaltered, probably due to the dominant presence of O–H bonds masking weaker contributions at the wavelengths where N–H vibrations typically appear.

To summarise, the optimised hydrogel film was realised starting from a 0.1 M KNO_3_ aqueous solution containing 0.7% agarose and 90 mM NH_3_. The mixture was applied by dip-coating and, after gelation at room temperature, the hydrogel film was immersed in pure glycerol for 10 s. 

### 3.3. NH_3_ Gas Sensor Response

**Figure 4:**
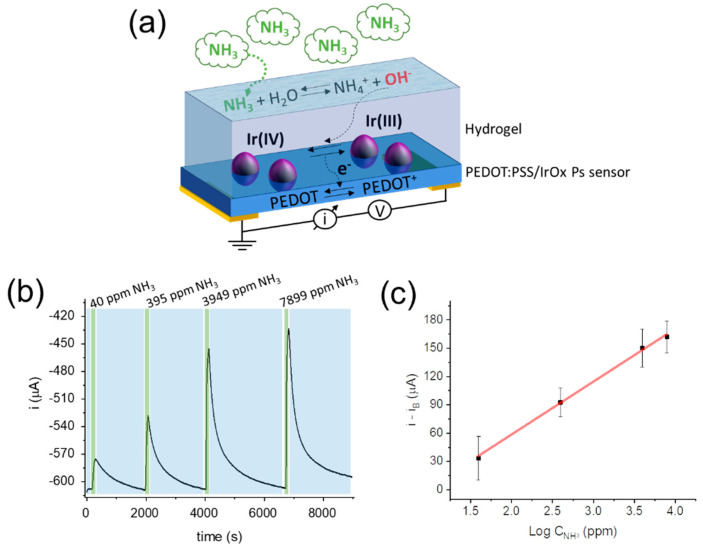
(**a**) Schematic of the optimised two-terminal gas sensor working principle. (**b**) Current vs. time response of the NH_3_ sensor and (**c**) calibration curve (R^2^ = 0.997) obtained from three repeated measurements. NH_3_-rich air streams were delivered for 100 s (green shadows) and alternated to air flow until full baseline recovery (blue shadows). Flow rate 2 L min^−1^; V_app_ = −200 mV.

The agarose/NH_3_/gly hydrogel is an essential part of the solid/gas interface composing the NH_3_ gas sensor based on PEDOT:PSS/IrOx Ps. In fact, it provides the aqueous-like environment where electrochemical gating takes place through a pH-mediated mechanism. A schematic of the sensor working principle is given in Figure 4a. 

As NH_3_ gas molecules reach the hydrogel surface, they diffuse inside the hydrogel structure and solvation occurs. Consequently, the acid-base equilibrium described in Equation (1) takes place and OH^−^ ions are released that increase the pH of the aqueous environment. This pH variation in the hydrogel corresponds to the chemical signal transduced by the PEDOT:PSS/IrOx pH-sensitive material. In particular, the pH affects the redox equilibrium involving the potentiometric transducer IrOx, i.e., the redox couple Ir^IV^/Ir^III^ present as IrO_2_ and Ir(OH)_3_ or IrOOH, which can be described by the Nernst equation [28]:

(2) $$E=E^{0}+\frac{RT}{F}\ln\left[H^{+}\right]$$

where E is the electromotive force generated by the spontaneous redox process, E^0^ is the standard reduction potential of the redox couple, R is the gas constant, T is the absolute temperature, and F is the Faraday constant. As a result of a local pH increase within the hydrogel, the oxidation of Ir(III) species to Ir(IV) is favoured and an electron is extracted from the IrOx Ps. Thanks to the intimate contact between IrOx Ps and PEDOT:PSS, the electron is directly injected into the polymer film where PEDOT^+^ reduction takes place. Due to its p-doped character, the organic semiconductor decreases its conductivity and, if a small ΔV is applied across the two-terminal sensor, the generated current decreases as well. 

The response of the optimised two-terminal sensor (Figure 4c) to gaseous NH_3_ was investigated using the calibrated stripping system (Figure S1). A ΔV of −200 mV was applied between the two terminals of the sensor, and the generated current was recorded over time, while the NH_3_-rich air streams with controlled NH_3_ concentration were delivered to the sensing chamber for 100 s at 2 L min^−1^ and alternated with air streams until baseline recovery (Figure 4b and Figure S14).

In accordance with the transduction mechanism discussed above, a sharp current decrease was observed right after the NH_3_-rich air flow entered the detection chamber, suggesting the rapid variation of the hydrogel pH upon gaseous NH_3_ exposure and the consequent redox interaction between the IrOx Ps and the PEDOT:PSS film. The signal reached a stable value after 100 s, and thus, the NH_3_ was removed from the chamber. The fundamental role of IrOx Ps during the detection was demonstrated with a blank experiment (Figure S15). In fact, if a hydrogel-coated PEDOT:PSS film is used without IrOx Ps, a current drift is recorded, and the baseline is not recovered due to the lack of a stable potentiometric system allowing pH transduction. The current variation was chosen as the analytical signal, and a correlation with the logarithm of the analyte concentration was found during repeatability measurements (Figure 4c, R^2^ = 0.997, N = 3). The sensitivity of the two-terminal gas sensor was (58 ± 9) μA decade^−1^, with a linear response to the NH_3_ concentration spanning three orders of magnitude and a response time (t_90_) of (87 ± 9) s. The LoD and LoQ (limit of quantification) values were 8 and 9 ppm, respectively. Although these results demonstrate that the sensor is suitable for gaseous NH_3_ quantification in air streams at ambient conditions, the long recovery time (t_10_) of (13 ± 2) × 10^2^ s renders this approach scarcely applicable to real experimental conditions.

**Figure 5:**
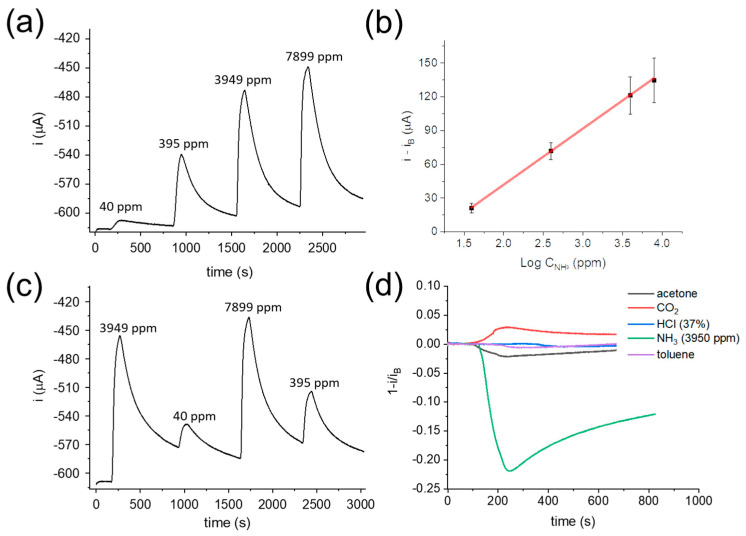
(**a**) Current vs. time response of the NH_3_ sensor and (**b**) calibration curve (R^2^ = 0.999) obtained from three repeated measurements performed without waiting for baseline recovery. NH_3_-rich air streams were delivered for 100 s and alternated to air flow for 600 s. Flow rate 2 L min^−1^; V_app_ = −200 mV. (**c**) Sensor response recorded upon exposure to random NH_3_ concentrations. (**d**) Sensor response recorded upon exposure to common gaseous compounds.

The performance of the two-terminal gas sensor was thus evaluated using experimental conditions that were closer to a real application. First, the sensing capability was verified without waiting for the baseline recovery by delivering the humid air flow for a fixed period of 600 s after each NH_3_ gas exposure. The real-time sensor response and correspondent calibration curve (R^2^ = 0.999, N = 3) are reported in Figure 5a,b. 

The resulting sensitivity of (49 ± 10) (mean ± standard deviation obtained from three independent calibrations) μA decade^−1^ is statistically comparable with the one calculated from the baseline recovery measurements, while the LoD (15 ppm) and LoQ (17 ppm) values are slightly higher when the baseline is not recovered. Overall, the relative standard deviation (RSD%) calculated on the sensitivity of four different sensors was 14%. Moreover, the sensor response was assessed during exposure to random NH_3_ concentrations, i.e., not increasing as described before (Figure 5c), highlighting the reliability of the two-terminal sensor and without any loss in sensitivity ((51 ± 5) μA decade^−1^). It is worth noting that the developed sensor here was always employed in combination with a bulky stripping system due to the need to generate gas streams with known NH_3_ concentrations, which are essential during sensor calibration. However, NH_3_ detection in a real environment will only require the contact between the gas under investigation and the sensor, which could be worn as a part of the personal protective equipment thanks to its light weight and small size. In such conditions, the reliability and stability of the gas sensor response should be guaranteed as we demonstrated that the calibrations derived from current variations evaluated from the initial baseline or without baseline recovery are statistically equal, and no hysteresis effect was observed.

Finally, an interference study was carried out by exposing the sensor to common gaseous chemicals, including saturated acetone and toluene vapours, pure CO_2_ stream, and concentrated HCl vapours (gas concentration: 15% *v*/*v*) (Figure 5d). Considering the two volatile organic compounds, toluene is scarcely water soluble and did not significantly affect the sensor response. Conversely, acetone is water soluble and generated a small current decrease, which might be due to its reactivity with NH_3_ to form acetone imine within the hydrogel, with the consequent pH variation due to the NH_3_ consumption. As regards HCl and CO_2_, both of them are water soluble, but while the first one is a strong and volatile inorganic acid, the latter is a weak acid when dissolved in water. In the case of HCl, the buffering capability of the NH_3_-rich hydrogel is probably sufficient to keep the pH unaltered upon exposure to the acid stream. In contrast, the high amount of pure CO_2_ delivered to the detection chamber caused an appreciable current increase associated with a local acidification of the hydrogel pH. However, all the investigated compounds produced less than a 5% current variation in the sensor response despite their high concentrations, demonstrating the high selectivity of the optimised device. 

### 3.4. Development of a Flexible Gas Sensor for Wearable Applications

Among the major advantages displayed by the developed hydrogel-modified PEDOT:PSS/IrOx Ps gas sensor for ammonia detection, the low cost, the low power consumption (around 0.1 mW), the wide concentration range, and the compatibility with ambient conditions make this device suitable for wearable applications in the field of occupational safety and health. In fact, on one hand the simple architecture leads to low material costs for production, and the small power consumption meets the requirements of portable electronic devices. On the other hand, the operative range of the sensor, including the limits set by the National Institute for Occupational Safety and Health (NIOSH) for daily and short-term exposure to NH_3_, and the robustness against humidity changes at room temperature suggest a possible integration within smart clothes, work suits, and accessories. 

**Figure 6:**
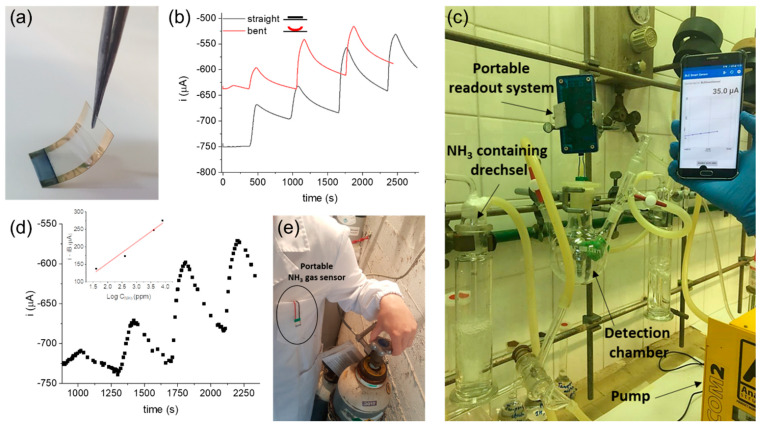
(**a**) Picture of the flexible two-terminal sensor. (**b**) Current vs. time response of the flexible NH_3_ sensor in the straight and bent configurations. (**c**) Picture of the flexible gas sensor detecting NH_3_ interfaced with a portable readout electronic system, which was wirelessly connected to a smartphone application via Bluetooth. (**d**) Real-time response of the flexible sensor in the portable configuration. Inset: calibration curve (R^2^ = 0.953). (**e**) Picture of the gas sensor placed in the breast pocket of a lab coat, simulating a real-life use for personal safety.

The two-terminal gas sensor was therefore fabricated on a flexible and transparent polyethylene terephthalate foil (Figure 6a), following the procedure optimised for the glass substrate. The performance of the flexible gas sensors was assessed in two configurations, e.g., with the sensor straight and bent at a 60° angle. The real-time responses are reported in Figure 6b. Using the experimental conditions optimised in Section 3.3, the flexible device retained almost the same sensitivity to NH_3_ ((60 ± 8) μA decade^−1^ and (49 ± 5) μA decade^−1^ for the straight and the bent configurations, respectively). 

A *t* test was carried out confirming that the two sensitivities are statistically equivalent within a confidence level of 95%, thus suggesting that the sensor response is unaffected by the applied mechanical deformations. In order to verify the compatibility with portable readout electronics, the flexible gas sensor was connected to a handheld reader powered by a coin cell battery and wirelessly connected to a smartphone for data acquisition (Figure 6c). The real-time response of the sensor working in the portable configuration is reported in Figure 6d; it shows a sensitivity of (60 ± 8) μA decade^−1^ during NH_3_ gas detection, with no performance loss with respect to the standard working conditions. These results demonstrate the applicability of the two-terminal gas sensor to real-life uses for personal safety and to the design of smart work suits (Figure 6e). Nevertheless, the influence of temperature on the response of the gas sensor should be considered if dealing with outdoor testing. In fact, while the stability of the IrOx Ps/PEDOT:PSS two-terminal sensor calibration was assessed upon temperature variations ranging from 20 to 40 °C in aqueous environment [28], potential effects on gas solubility within the hydrogel film should be investigated.

Table 1 summarises the analytical performance of the developed two-terminal NH_3_ gas sensor and other literature reports concerning flexible gas sensors based on conducting polymer composites. To allow a direct comparison, the sensitivity is expressed as ΔR/R_0_ % ppm^−1^, where ΔR and R_0_ refer to the variation of the analytical signal and the baseline signal of the sensor, respectively. 

Considering their analytical performance, these NH_3_ sensors are applicable for environmental monitoring [23,42,43,44] as well as food quality and safety monitoring [45,46,47], all of them sharing the essential, highly integrated chemoresistor architecture that adapts well to wearable electronics. However, PANi is the most used transducer, and the sensing performance of PANi-based sensors are usually comparable to each other, with sensitivities lower than 1% ppm^−1^ and a linear response ranging from units to hundreds of ppm. However, the combination of PANi with PTS or MoS_2_ improves the sensitivity [46] or extends the linear response to lower concentrations [44], respectively. Among other conductive polymers, PEDOT:PSS takes advantage of high chemical stability and conductivity, even if the detection capability is lower than that of PANi. Therefore, the transduction exploits the peculiar morphology of nanowires [45] or the change of resistance at the PEDOT:PSS/Ag NWs interface [47]. These sensors operate at low concentration (below 25 ppm) and with a sensitivity of about 1% ppm^−1^. While retaining the simple two-terminal structure, the PEDOT:PSS/IrOx Ps sensor differs in the transducing principle as it benefits from the robustness of a potentiometric-like mechanism and is compatible with low energy supply systems. The signal can be easily acquired with portable and wireless electronics, while most of the sensors in the literature have only been demonstrated in a wired configuration. Moreover, a wider concentration range and shorter response time are achieved with respect to the most sensitive gas sensor based on PTS/PANi (112 s) [46].

**Table 1:** Summary of flexible NH_3_ gas sensors performances, based on conducting polymers reported in the literature.

Sensing Material	Substrate	Connectivity	Range (ppm)	Sensitivity (% ppm^−1^)	Ref.
MWCNTs/PANi/PU foam	PDMS	Wired	5–200	0.65	[42]
PEDOT: PSS NWs	PET	BT	0.75–6	0.25	[45]
PTS/PANi	PET	NFC	5–200	45	[46]
PANi/PAN	Textile	Wired	10–2000	N/A	[43]
C-PPy NPs	Plastic	Wired RFID tag	0.1–25	0.2	[23]
PEDOT: PSS/Ag NWs	PET	Wired	0.5–25	1.40	[47]
PANI/MoS2	PDMS	Wired	0.05–30	N/A	[44]
PEDOT: PSS/IrOx Ps	PET	BT	17–7899	0.8	this work

MWCNTs, multiwalled carbon nanotubes; PU, polyurethane; NWs, nanowires; PTS, p-toluene sulfonate hexahydrate; PAN, polyacrylonitrile; C-PPy, carboxylated Polypyrrole.

## 4. Conclusions

An innovative, two-terminal gas sensor for NH_3_ detection was designed here that includes a hydrogel interface that assists the detection mechanism based on electrochemical gating. The working principle of the proposed device originates from the potentiometric pH transducer IrOx, which is embedded in the form of particles within a thin film of organic semiconductor and responds to local pH variations in the hydrogel, thus modulating the doping state of the semiconductor. On one hand, the electrochemically gated sensor takes advantage of a more robust sensing mechanism but retains the essential structure of a chemiresistor upon application of a small potential difference. Indeed, the combination of robustness, low power consumption (around 0.1 mW), remarkable analytical performance, and softness makes this device suitable for wearable applications. On the other hand, the hydrogel interface, where the gaseous analyte must reversibly absorb and dissolve, producing pH variations, is actually the key component of the sensor structure. In fact, its acid-base properties, chemical structure, and morphology can be finely optimized to achieve the desired selectivity towards different volatile compounds, thus providing the sensing architecture developed here with remarkable versatility. In conclusion, the novelty of the two-terminal gas sensor may pave the way for the design of lightweight multivariable sensing platforms for wearable applications.

## Supplementary Materials

The following are available online at https://www.mdpi.com/article/10.3390/s21237905/s1, Figure S1: ammonia stripping system, Figure S2: complexation of NH_3(g)_ with H_3_BO_3_ to form (NH_4_)B(OH)_4_, Figure S3: titration curve of (NH_4_)B(OH)_4_ with 0.01000 M HCl, Figure S4: calibration of the stripping system, Table S1: correlation between [NH_3_]_(l)_ and [NH_3_]_(g)_ obtained after 100 s stripping at a flow rate of 2.00 L min^−1^, Figure S5: PEDOT:PSS/IrOx Ps, two-terminal pH sensor response in solution, Figure S6: comparison between pristine and glycerol-treated agarose hydrogel on PEDOT:PSS films, Figure S7: comparison between two-terminal PEDOT:PSS/IrOx Ps sensors modified with 1 mM phosphate buffer (pH 7.00) and 0.1 M KNO_3_ containing hydrogels, Figure S8: effect of agarose content, Figure S9: repeated exposure of a PEDOT:PSS/IrOx Ps sensor, Figure S10: qualitative evaluation of pH variations in a 0.7% agarose in 0.1 M KNO_3_ hydrogel by adding a set of pH dyes to the hydrogel composition, Figure S11: response of two-terminal PEDOT:PSS/IrOx Ps sensors modified with (a) 0.4 and (b) 90 mM NH_3_ containing hydrogels, Figure S12: comparison of the analytical performance obtained from two-terminal PEDOT:PSS/IrOx Ps sensors modified with 0.4, 1, 10, or 90 mM NH_3_ containing hydrogels, Figure S13: IR characterisation, Figure S14: zoom of Figure 4b, Figure S15: exposure of two-terminal devices, with and without IrOx Ps and hydrogel, to a NH_3_-rich air stream.
