# Supplementary material for: A Wearable Electrochemical Gas Sensor for Ammonia Detection
Source: Sensors (Basel). 2021 Nov 27;21(23):7905. doi: 10.3390/s21237905 (PMC8659774; doi:10.3390/s21237905)
Supplement: Supplementary file 1 [file sensors-21-07905-s001.zip › sensors-1469594-supplementary.pdf]

sensors

MDPI

A Wearable Electrochemical Gas Sensor for Ammonia Detection

Martina Serafini [1], Federica Mariani [1,*], Isacco Gualandi [1,*], Francesco Decataldo [2], Luca Possanzini [2], Marta Tessarolo [2], Beatrice Fraboni [2], Domenica Tonelli [1] and Erika Scavetta [1]

[1] Dipartimento di Chimica Industriale "Toso Montanari", Università di Bologna, Viale del Risorgimento 4, 40136 Bologna, Italy; martina.serafini6@unibo.it (M.S.); domenica.tonelli@unibo.it (D.T.); erika.scavetta2@unibo.it (E.S.)

[2] Dipartimento di Fisica e Astronomia, Università di Bologna, Viale Berti Pichat 6/2, 40127 Bologna, Italy; francesco.decataldo2@unibo.it (F.D.); luca.possanzini2@unibo.it (L.P.); marta.tessarolo3@unibo.it (M.T.); beatrice.fraboni@unibo.it (B.F.)

* Correspondence: federica.mariani8@unibo.it (F.M.); isacco.gualandi2@unibo.it (I.G.)

Supplementary Materials:

Ammonia stripping system assembly and calibration

A pump for air sampling was used to apply a vacuum to the pipes and drive the gas stream throughout the sensing chamber (flow rate of 2 L min^{-1}). Humid air and gaseous NH_3 streams were alternated using a three-way valve connected to Drechsel bottles 1 and 2, containing D.I. water and NH_3 aqueous solutions of known concentration, respectively. Active carbon cartridges were employed to avoid contamination from any organic species traces in air. A third Drechsel bottle containing D.I. water was positioned before the pump to re-absorb NH_3 from the gas stream (Figure S1).

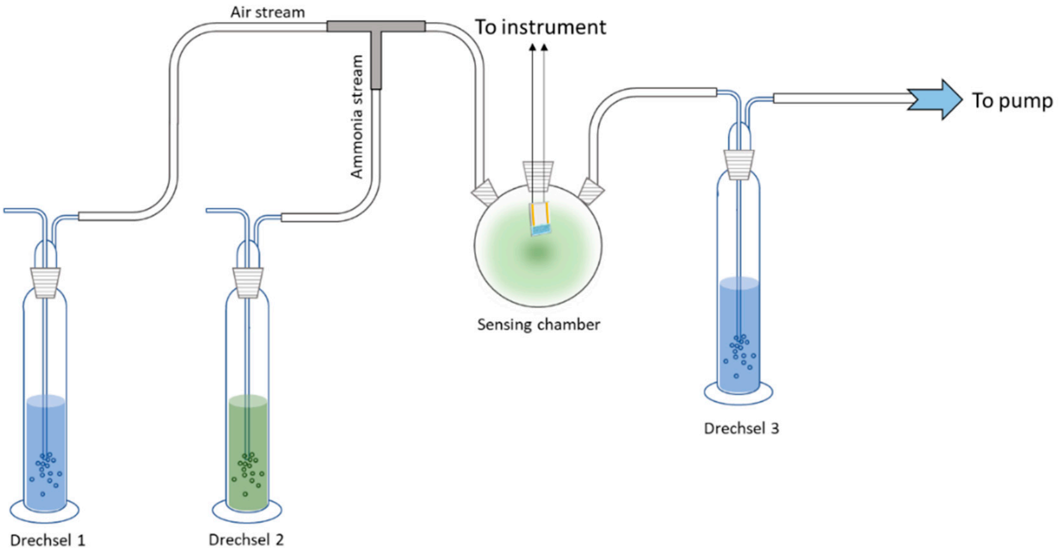

Figure S1. Ammonia stripping system.

The system was calibrated by means of a validated analytical method (titration) to establish the exact correlation between $[NH_3]_{(l)}$ and $[NH_3]_{(g)}$ delivered to the sensing chamber. For this, a modified version of the Kjeldahl method was chosen [1] that consisted of two steps: (i) complexation of $NH_{3(g)}$ with H_3BO_3 to form $(NH_4)B(OH)_4$ and (ii) potentiometric titration with standardized HCl. First, 25 mL of standardised NH_3 aqueous solution (0.01000, 0.04000, or 0.1000 M) was put in a Drechsel bottle, which was in turn connected to a second one containing 25 mL of 0.1 M H_3BO_3 and attached to the pump (Figure S2).

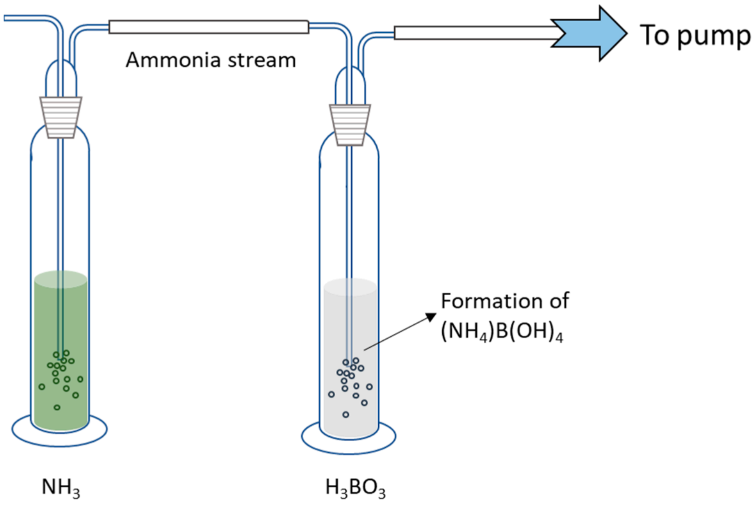

Figure S2. Complexation of $NH_{3(g)}$ with H_3BO_3 to form $(NH_4)B(OH)_4$.

NH_3 stripping from the aqueous solution was performed at a flow rate of 2 L min^{-1} for 12.5 min, thus ideally delivering 25 L of NH_3(g) to the boric acid solution. In diluted solutions of H_3BO_3, ammonia was dissolved as follows:

$$NH_3 + H_2O \rightleftarrows NH_4^+ + OH^- \quad (1)$$

$$B(OH)_3 + OH^- \rightleftarrows B(OH)_4^- \quad (2)$$

leading to its complexation in the form of ammonium tetrahydroxyborate $(NH_4)B(OH)_4$.

The solution of H_3BO_3 and $(NH_4)B(OH)_4$ was quantitatively transferred into a flask and a potentiometric titration with standardized 0.01000 or 0.1000 M HCl was carried out using a combined glass electrode connected to a pH meter. In order to visually identify the titration endpoint, a few drops of an ethanol solution containing methyl red (0.03% *w*/*v*) and methylene blue (0.1% *w*/*v*), known as Tashiro indicator (pH range of color change from green to blue: 6.6–4.4), were added to the sample. Upon addition of HCl, the following reaction takes place until complete consumption of the ammonium complex:

$$HCl + (NH)_4B(OH)_4 \rightarrow NH_4Cl + H_3BO_3 + H_2O \quad (3)$$

An example of the titration curve is provided in Figure S3.

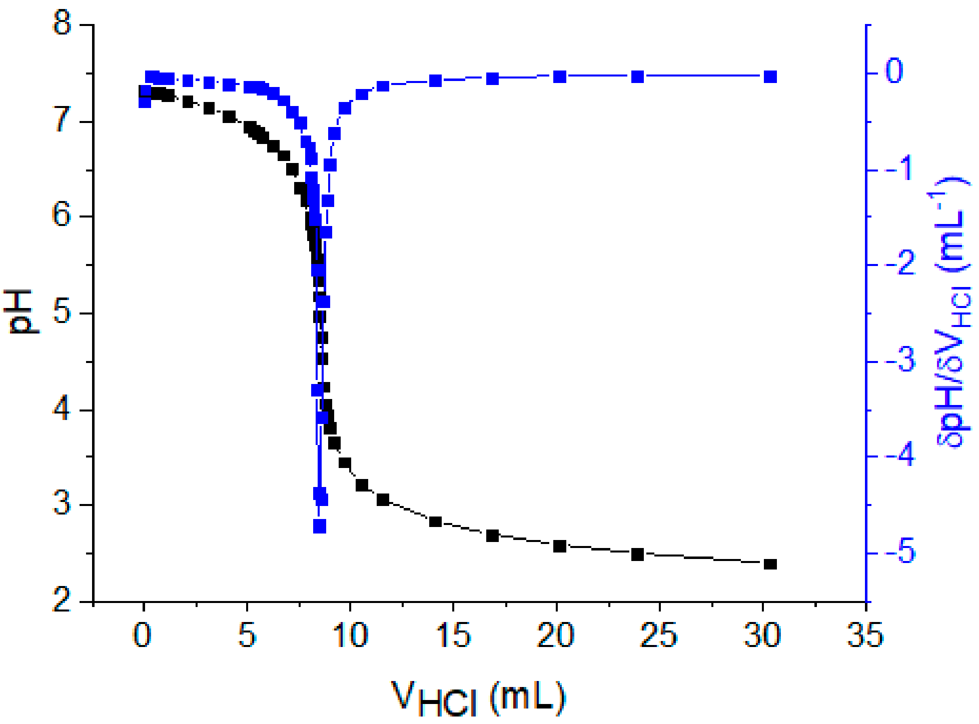

Figure S3. Titration curve of $(NH_4)B(OH)_4$ with 0.01000 M HCl following 0.01000 M NH_3 aqueous solution stripping and bubbling into 25.0 mL of a 0.1 M H_3BO_3 solution at a flow rate of 2.00 L min^{-1} for 12.5 min.

As all the involved reactions have 1:1 stoichiometry, the moles of HCl consumed at the titration endpoint are equivalent to the moles of $NH_{3(g)}$ obtained from the stripping process that were initially flushed into the H_3BO_3 solution. Consequently, a linear correlation between $[NH_3]_{(l)}$ and $[NH_3]_{(g)}$ was found, as shown in Figure S4.

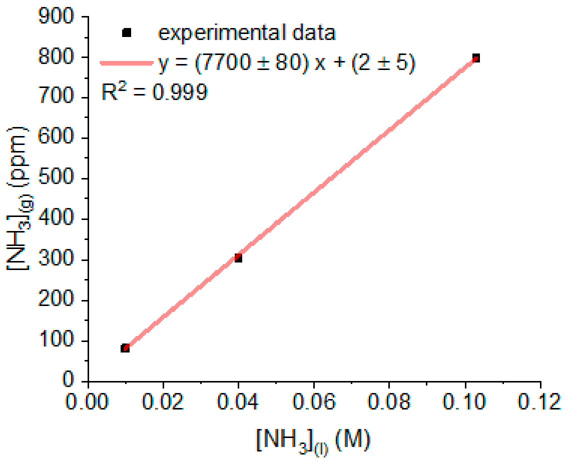

Figure S4. Calibration of the stripping system. Correlation between $[NH_3]_{(l)}$ and $[NH_3]_{(g)}$ obtained after 12.5 min stripping at a flow rate of 2.00 L min^{-1}.

As the y-intercept value is negligible considering the associated standard error, the equation characteristic of this stripping system can be approximated to y = mx in accordance with Henry's law. The efficiency of the stripping system was estimated in the range of 45–50%, including eventual head losses.

Finally, integral calculations were conducted in order to determine $[NH_3]_{(g)}$ as obtained after 100 s stripping at 2.00 L min^{-1}, i.e., in the experimental conditions selected

for the measurements with the two-terminal sensor. The results are summarised in Table S1.

Table S1. Correlation between $[NH_3]_{(l)}$ and $[NH_3]_{(g)}$ obtained after 100 s stripping at a flow rate of 2.00 L min^{-1}.

**$[NH_3]_{(l)}$ (M)**	**$[NH_3]_{(g)}$ (ppm)**
4.00×10^{-4}	4.00
4.00×10^{-3}	40.0
4.00×10^{-2}	395
4.00×10^{-1}	3.95×10^{3}
8.00×10^{-1}	7.90×10^{3}
1.00	9.87×10^{3}

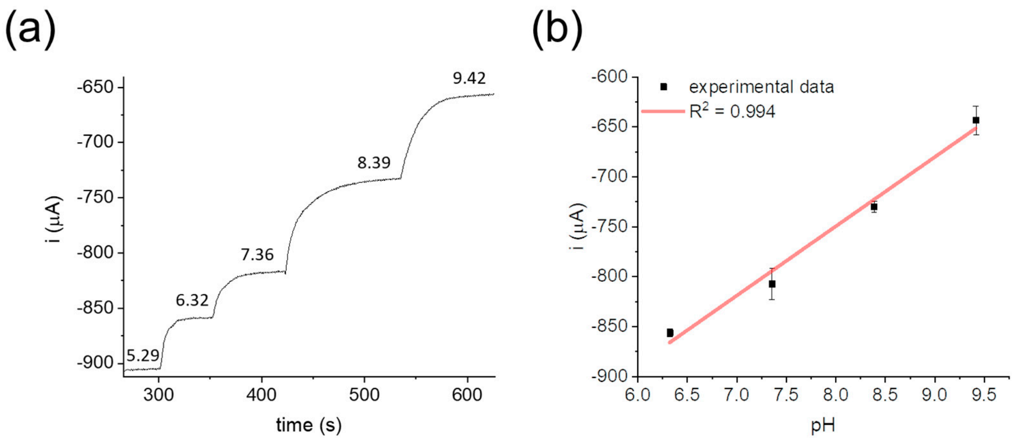

Figure S5. PEDOT: PSS/IrOx Ps, two-terminal pH sensor response in solution. (**a**) Current vs. time response recorded in buffer solution upon addition of 1 M KOH. V_{app} = −200 mV. (**b**) Calibration curve obtained from five independent measurements carried out with the same pH sensor.

Optimization of the hydrogel composition

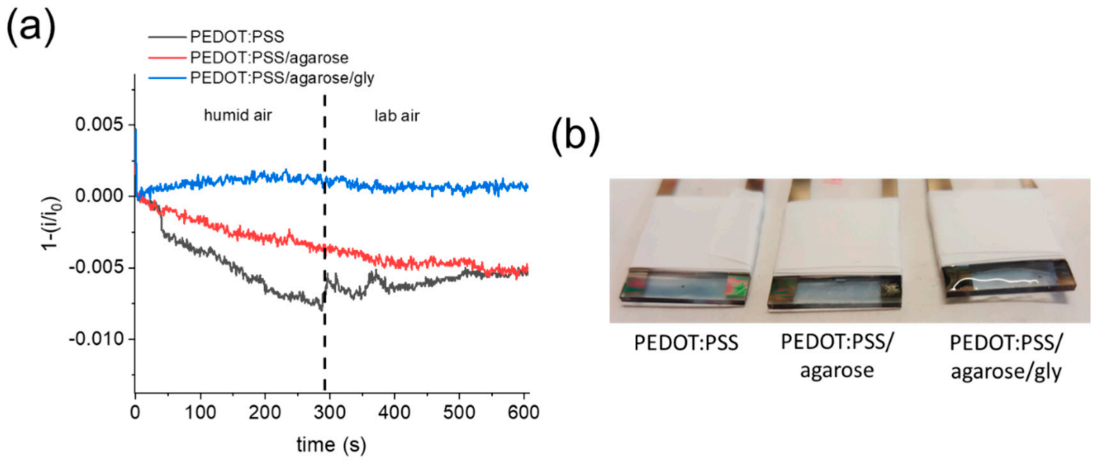

Figure S6. Comparison between pristine and glycerol-treated agarose hydrogel on PEDOT: PSS films (bare PEDOT: PSS used as control). (**a**) Normalised currents recorded from devices placed inside the detection chamber of the stripping system, during alternate exposure to saturated and unsaturated air streams at a flow rate of 2.00 L min^{-1}. V_{app} = −200 mV. (**b**) Picture of the three films right after the experiment.

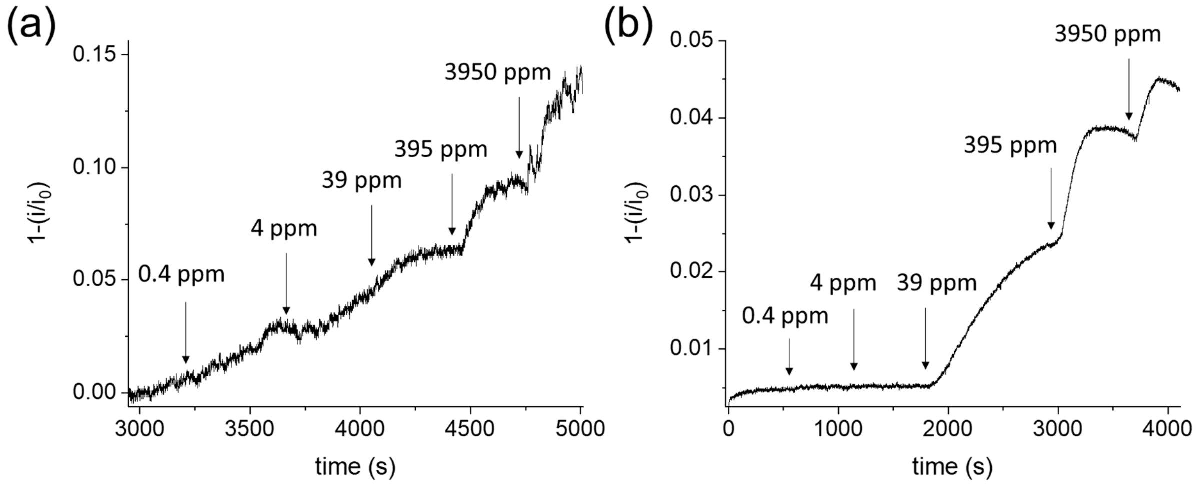

Figure S7. Comparison between two-terminal PEDOT: PSS/IrOx Ps sensors modified with (**a**) 1 mM phosphate buffer (pH 7.00) and (**b**) 0.1 M KNO_3 containing hydrogels during gaseous NH_3 detection. NH_3-rich streams of increasing concentration were delivered for 100 s to the detection chamber and alternated to humid air at a flow rate of 2.00 L min^{-1}. V_{app} = −200 mV.

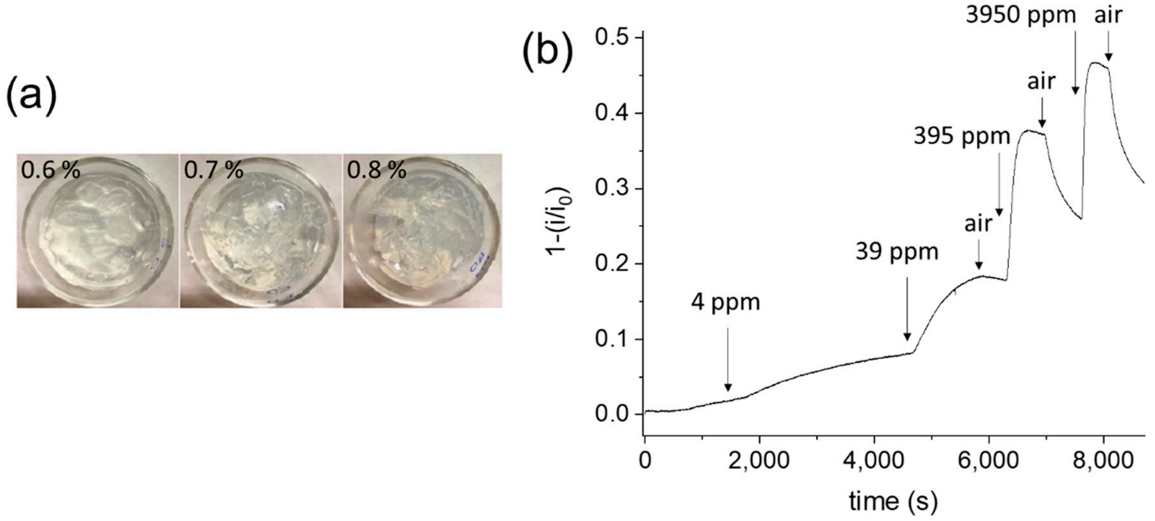

Figure S8. Effect of agarose content. (**a**) From left to right: hydrogels made up with 0.6% (partial gelation), 0.7% (full gelation) and 0.8 % (full gelation) agarose in 0.1 M KNO_3. (**b**) Response of a two-terminal PEDOT: PSS/IrOx Ps sensor modified with the 0.7% agarose hydrogel in 0.1 M KNO_3 after gly treatment. NH_3-rich streams of increasing concentration were delivered for 100 s to the detection chamber and alternated to humid air at a flow rate of 2.00 L min^{-1}. V_{app} = −200 mV.

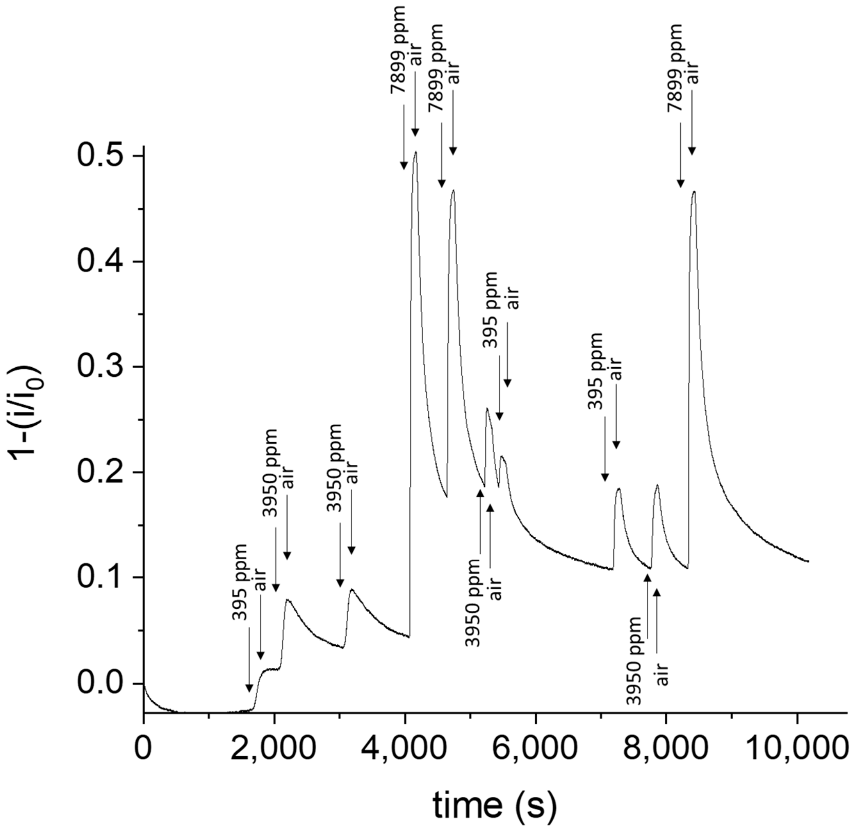

Figure S9. Repeated exposure of a PEDOT: PSS/IrOx Ps sensor modified with a glycerol-treated, 0.7% agarose hydrogel in 0.1 M KNO_3 to concentrated NH_3 streams delivered for 100 s to the detection chamber and alternated to humid air at a flow rate of 2.00 L min^{-1}. V_{app} = −200 mV.

Indicator	pKa	Color change
Phenolphtalein	9.4	C - R
Methyl Red	4.9	R - Y
Thymol Blue	1.7<br>8.9	R - Y<br>Y - B
Bromothymol Blue	7.1	Y - B

C = colorless; R = red; Y = yellow; B = blue.

Control (agarose hydrogel)

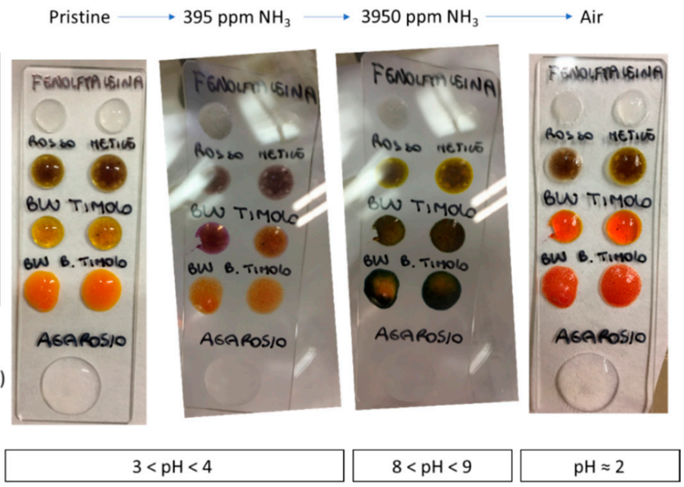

Figure S10. Qualitative evaluation of pH variations in a 0.7% agarose in 0.1 M KNO_3 hydrogel by adding a set of pH dyes to the hydrogel composition. Color changes were observed once the glass slide was placed inside the detection chamber and exposed to NH_3 and air streams.

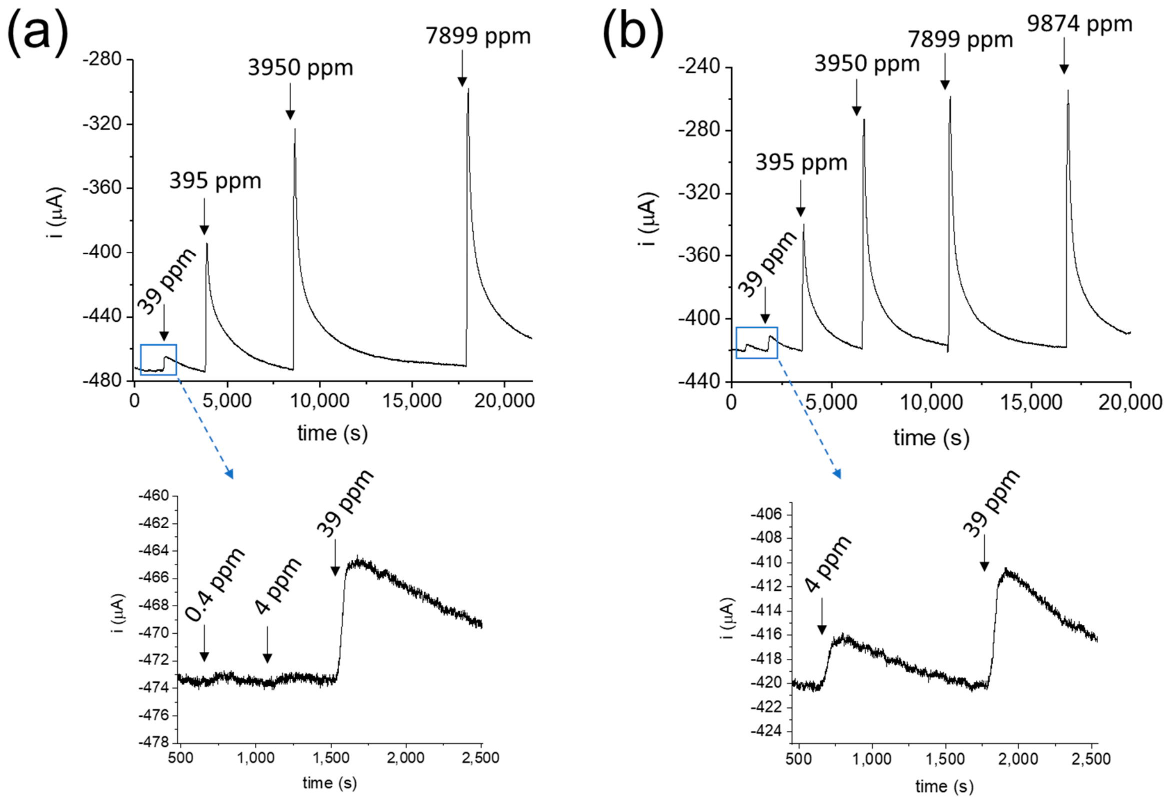

Figure S11. Response of two-terminal PEDOT: PSS/IrOx Ps sensors modified with (**a**) 0.4 and (**b**) 90 mM NH_3-containing hydrogels. NH_3-rich streams of increasing concentration were delivered for 100 s to the detection chamber and alternated to humid air at a flow rate of 2.00 L min^{-1}. V_{app} = −200 mV.

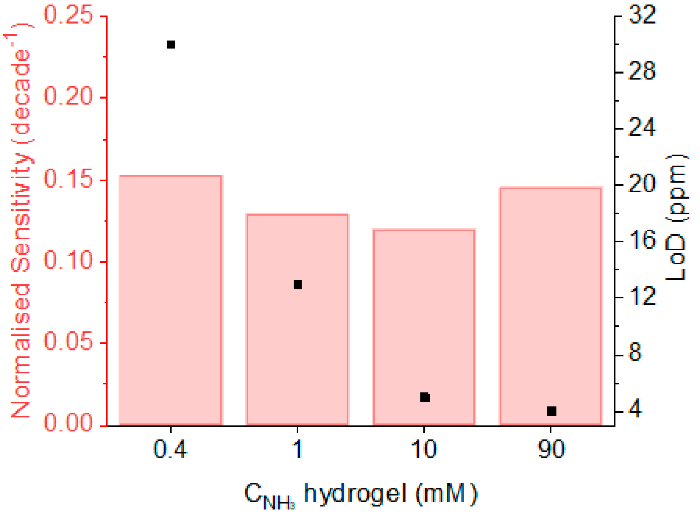

Figure S12. Comparison of the analytical performance obtained from two-terminal PEDOT: PSS/IrOx Ps sensors modified with 0.4, 1, 10, or 90 mM NH_3-containing hydrogels.

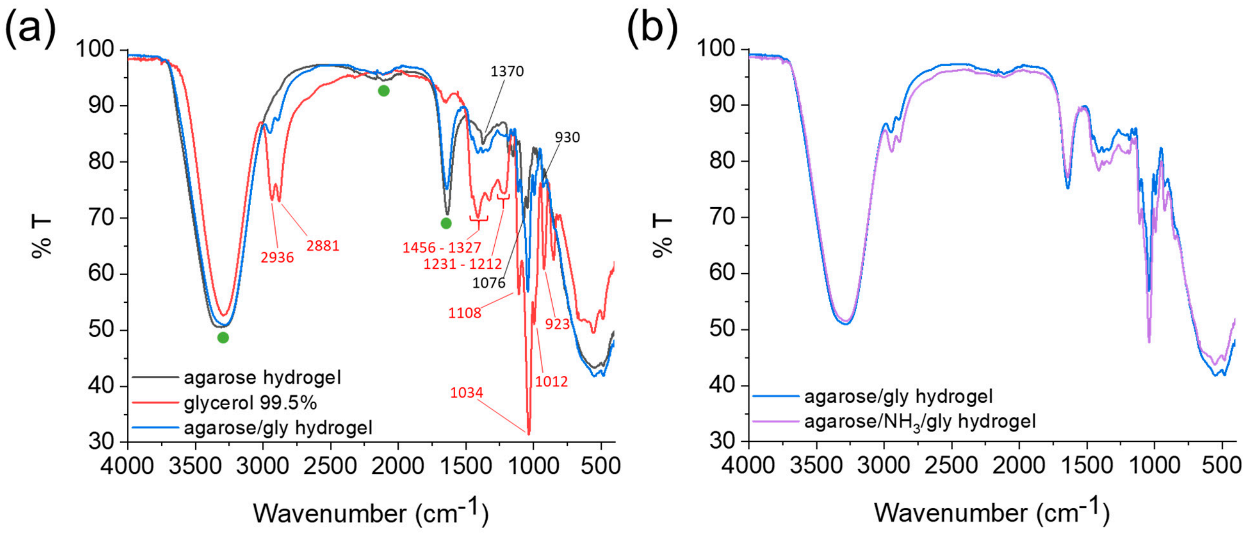

Figure S13. IR characterization. (**a**) Spectra of agarose hydrogel, glycerol, and agarose/gly hydrogel. Characteristic water peaks are highlighted by green circles. (**b**) Spectra of agarose/gly and the optimized agarose/NH_3/gly hydrogels.

All the IR spectra are characterised by the strong fingerprint of –OH groups, which are due to water presence and to the hydroxyl functional groups in both the agarose and the glycerol. In particular, the peaks at 3500, 1635, and 2120 cm^{-1} are typical of O–H stretching, O–H–O scissors-bending, and the coupling of the latter with a librational motion of H-bonded H_2O molecules, respectively, and are labelled with green circles in Figure S12a [2]. The characteristic peaks of agarose appear at 1370 cm^{-1}, which is assigned to ester-sulfate link vibrations, and at 1076 and 930 cm^{-1}, which are associated with the glycosidic bond vibrations [3,4]. The presence of residual sulfate groups in the agarose structure has been discussed in the literature [5] and is in accordance with the spontaneously acidic pH of the hydrogel. As regards glycerol, the bimodal peak at 2936–2881 cm^{-1} is assigned to asymmetric vibrations of the –CH_2 groups, while the C–H bending and deformation vibrations appear at 1456, 1411, and 1327 cm^{-1}. The peaks at 1231 and 1212 cm^{-1} are associated to C–O stretching, and the peak at 1108 cm^{-1} is assigned to C–C stretching, while the peaks at 1034 and 1012 cm^{-1} are ascribed to C–H deformation and C–C stretching. Bending of the –OH group is seen at 923 cm^{-1} [6]. The spectrum of the agarose/gly hydrogel (blue line) clearly results from the combination of its components (black and red curves). The IR spectrum of the agarose/NH_3/gly hydrogel optimized in this work is shown in Figure S12b. While all the peaks discussed above that are characteristic of the NH_3-free hydrogel are still present, the inclusion of NH_3 in the hydrogel structure does not lead to any apparent change. In fact, no additional peaks at 3500–3300 cm^{-1} (N–H stretching) and 1650–1580 cm^{-1} (N–H bending) are detected. This could be due to the prominent O–H features masking weaker contributions at those wavelengths.

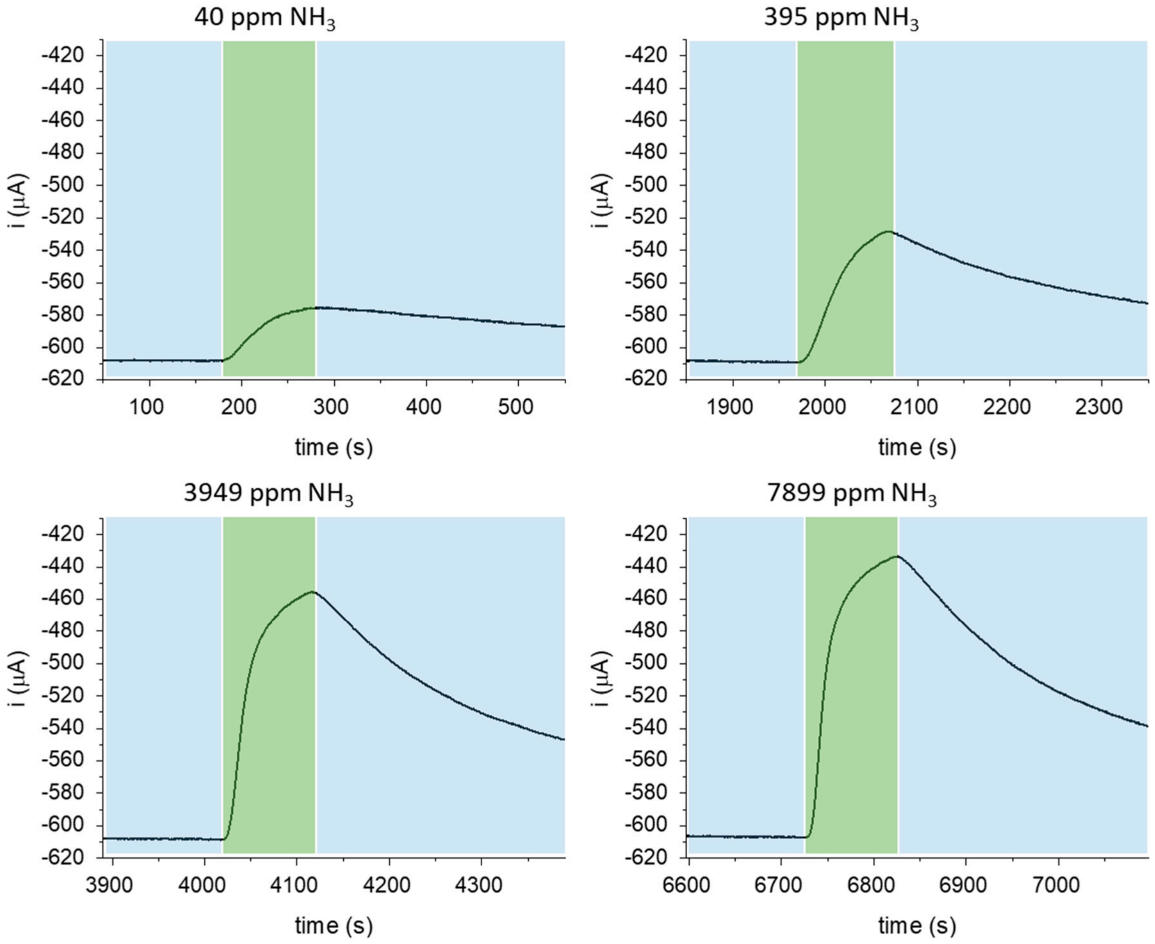

Figure S14. Zoom of Figure 4b illustrating the recorded current variations vs. time in response to the different NH_3-rich streams delivered for 100 s to the sensing chamber.

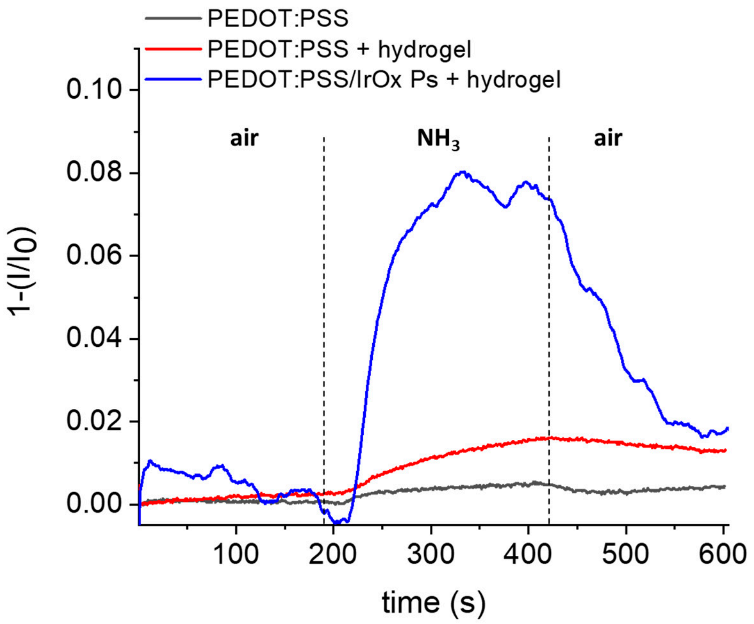

Figure S15. Exposure of two-terminal devices, with and without IrOx Ps and hydrogel, to a NH_3-rich air stream for 100 s at a flow rate of 2.00 L min^{-1}. V_{app} = −200 mV.

References

1. Cruz, G. Boric Acid in Kjeldahl Analysis. *J. Chem. Educ.* **2013**, *90*, 1645–1648, doi:10.1021/ed4003767.
2. Verma, P.K.; Kundu, A.; Puretz, M.S.; Dhoonmoon, C.; Chegwidden, O.S.; Londergan, C.H.; Cho, M. The Bend+Libration Combination Band Is an Intrinsic, Collective, and Strongly Solute-Dependent Reporter on the Hydrogen Bonding Network of Liquid Water. *J. Phys. Chem. B* **2018**, *122*, 2587–2599, doi:10.1021/acs.jpcb.7b09641.
3. Mollet, J.-C.; Rahaoui, A.; Lemoine, Y. Yield, chemical composition and gel strength of agarocolloids of Gracilaria gracilis, Gracilariopsis longissima and the newly reported Gracilaria cf. vermiculophylla from Roscoff (Brittany, France). *J. Appl. Phycol.* **1998**, *10*, 59, doi:10.1023/A:1008051528443.
4. El-hefian, E.A.; Nasef, M.M.; Yahaya, A.H. Preparation and Characterization of Chitosan/Agar Blended Films: Part 1. Chemical Structure and Morphology. *E-J. Chem.* **2012**, *9*, 781206, doi:10.1155/2012/781206.
5. Armisen, R.; Galatas, F. Production, properties and uses of agar. Production and utilization of products from commercial seaweeds. *FAO Fish. Tech. Pap* **1987**, *288*, 1–57.
6. Kachel-Jakubowska, M.; Matwijczuk, A.; Gagoś, M. Analysis of the physicochemical properties of post-manufacturing waste derived from production of methyl esters from rapeseed oil. *Int. Agrophysics* **2017**, *31*, 175–182, doi:10.1515/intag-2016-0042.
